# Comparison of passive versus active transcutaneous bone anchored hearing devices in the pediatric population

**DOI:** 10.1186/s40463-022-00595-5

**Published:** 2022-11-12

**Authors:** Nael M. Shoman, Usman Khan, Paul Hong

**Affiliations:** Division of Otolaryngology-Head and Neck Surgery, QEII Health Sciences Centre,, Dalhousie University, 3184 Dickson Building, 5820 University Avenue, Halifax, NS B3H 2Y9 Canada

**Keywords:** Bone anchored hearing aid, Transcutaneous, Active, Passive, Pediatric

## Abstract

**Objective:**

Transcutaneous bone anchored hearing devices (BAHDs) were introduced in an effort to avoid potential complications associated with the abutment of percutaneous BAHDs. Transcutaneous BAHDs can be active or passive. While studies have demonstrated good outcomes with both, a direct comparison of audiological and clinical outcomes of these devices in the pediatric population has not yet been studied.

**Study design:**

Retrospective, multicenter study.

**Setting:**

Two tertiary academic centers.

**Methods:**

Between 2015 and 2019, all patients who received an active transcutaneous BAHD (Bonebridge, BB) at one center, and patients that received a passive transcutaneous BAHD (Attract, AT) at another center, were included in this study. Exclusion criteria included age > 18 years, and mixed hearing loss or single-sided deafness. Study outcomes included patient demographics, indications, complications and preoperative and one-year postoperative audiometric data.

**Results:**

Eighteen BB and eight AT patients met the inclusion criteria. The age range was 5–16 years. There were no significant differences in complication outcomes. Both devices demonstrated similar mean improvements in hearing thresholds at frequencies of 250 Hz (38 dB Active vs. 38 dB Passive), 500 Hz (34 dB vs. 42 dB), 1000 Hz (34 dB vs. 40 dB) and 2000 Hz (31 dB vs. 22 dB). The BB was significantly more effective at frequencies of 4000 Hz (28 dB vs. 7 dB) and 8000 Hz (29 dB vs. 6 dB) (*p* < 0.05).

**Conclusion:**

This is the first study comparing audiological outcomes between an active and a passive transcutaneous BAHD in the pediatric population. While both devices improved audiometric outcomes in the low and mid frequencies, the active BAHD demonstrated significantly better outcomes in the higher frequencies.

**Graphical Abstract:**

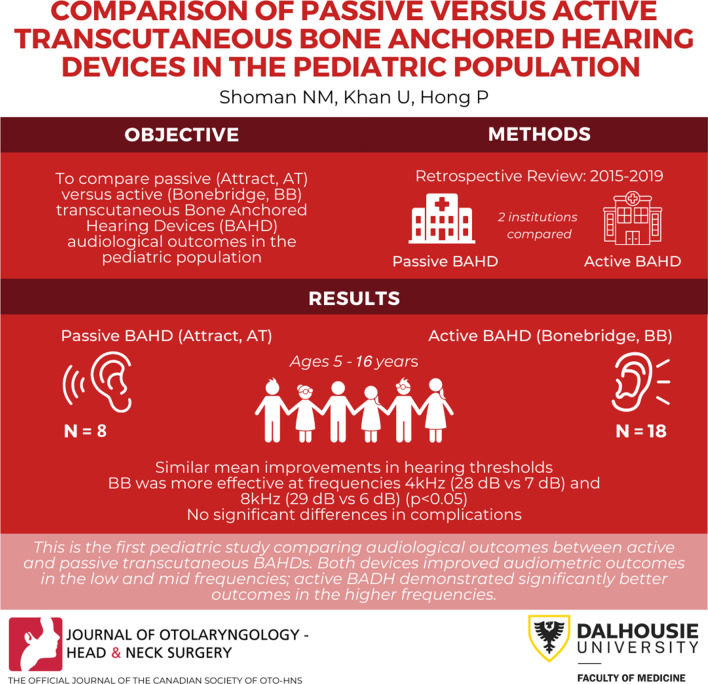

## Introduction

The concept of osseointegration, and the introduction of percutaneous bone anchored hearing devices (BAHDs), has provided many children with conductive hearing loss (CHL) a valuable option for auditory rehabilitation. Over time, the indications for their surgical placement have expanded to include more challenging mixed hearing loss (MHL) and single sided deafness (SSD) cases.

There are nevertheless important considerations in the pediatric population. Generally, complication rates with percutaneous BAHDs are higher in children [1–4]. There is a significantly higher rate of fixture failure, and this risk is higher in the very young, and in the first year following surgery [2, 4]. Soft tissue complications are more common in children as well [3, 5]. This is further complicated by the higher prevalence of underlying developmental delay and behavioural issues in this patient population, making regular maintenance of the abutment by the child and the care provider potentially more challenging [5].

Transcutaneous BAHDs have been introduced in an effort to avoid potential complications associated with an abutment. These devices are surgically placed under the skin, with a receiver worn externally which is held by a magnetic connection to the internal component. They are classified as passive and active. The passive transcutaneous BAHD Attract (AT, Cochlear, Sydney, Australia) was introduced in 2015, and received Health Canada approval for children 5 years and older. Concerns have been raised regarding soft tissue attenuation of the higher frequencies [6]. At the time of the study, there was only one transcutaneous active device that is Health Canada approved, Bonebridge (BB, MedEl, Innsbruck, Austria), which was approved in 2015 for children 5 years and older. The Cochlear Osia (Cochlear, Mölnlycke, Sweden) was more recently approved by Health Canada in 2020. To our knowledge, there are no published studies directly comparing the BB and AT devices in the pediatric population. The aim of this study as such was to present our clinical and audiological data in two cohorts of pediatric patients that received these two devices.

## Methods

Study design and methodology were reviewed and affirmed exempt by the Institutional Review Boards at Dalhousie University and the University of Saskatchewan. This was a multicenter study comparing the outcomes of a cohort of children that received the BB (BCI 601) at one center (Saskatoon), and a cohort that received the AT at another center (Halifax). In light of various system considerations, each center only implanted children with one particular device. Respectively, each cohort had all the surgeries done by the same surgeon using a standardized technique. The study design entailed the generation of a list of all children that received a BAHD from March 2015 till March 2019, and that list was then filtered to only those children that received a transcutaneous BAHD.

The indication for the device placement was then determined, and patients whose indication was that of SSD or MHL were excluded. This was done in an effort to better match the groups for more meaningful audiological comparisons. Overall inclusion criteria included patients < 18 years of age, normal bone conduction threshold limits (better than 20 dB HL at frequencies 500–8000 Hz), and stable inner ear function without hearing fluctuations. All children had formal candidacy evaluations completed, and patients and families were counselled regarding management options including the use of a percutaneous abutment, and surgical transcutaneous BAHD options. A demo trial with a soft band bone conduction hearing aid was part of this evaluation to assess objective and subjective improvement.

Clinical data retrieved on chart review included age at implantation, gender, any medical conditions, etiology of hearing loss, imaging workup and results, device surgical placement location, intraoperative details (such as the use of lifts in BB surgery), as well as intra- and peri-operative complications.

### Surgical description

All procedures were done under general anesthesia. For the BB, a standard postauricular incision was made followed by elevation of anterior and posterior skin flaps. An anteriorly based musculoperiosteal flap was raised. Mastoid drilling was initiated and continued in the sinudural angle, using the device template throughout until the fit was good to allow the demo template for the floating mass transducer (FMT) to completely sit within the drilled well, being deep enough to have the flanges flush with the surrounding bone. In cases where it was not possible to fit the FMT in the sinudural angle within the mastoid, supratemporal placement over the middle fossa dura was used instead. A posterior musculoperiosteal flap was raised to create a periosteal pocket for the receiver-transmitter. The implant system was then placed and the FMT secured using two 2-mm cortical bone fixation screws supplied by the manufacturer. In cases where these screws were not fixating, 2.4-mm emergency screws were used.

For the AT, the implant site was marked using the implant indicator, and a C-shaped incision was made 15 mm posterosuperior to the edge of the magnet. Following anterioinferior retraction of the soft tissue, the implant magnet template was used to ensure good positioning. Thereafter, a cruciate incision was made in the periosteum to expose the bone, and drilling was done in the standard fashion of placement of a 4 mm titanium screw. The implant post was then placed followed by attachment of the implant magnet.

### Audiological evaluation

Hearing thresholds were measured preoperatively as an ear-specific pure tone audiogram in the unaided condition as well as with bone conduction hearing aids, masking the non-test ear as needed. In children between five and nine years of age, conditioned play audiometry was used. In children over ten years, the response would be either hand raise or pressing a button. When possible, these included mean air conduction (AC) free-field testing at the frequencies of 250, 500, 1000, 2000, 4000, and 8000 Hz, as well as mean bone conduction (BC) at the frequencies of 500, 1000, 2000, and 4000 Hz. In children in whom this information was not attainable, visual reinforcement audiometric data was used. Likewise, word recognition data was obtained when feasible.

After 2–4 weeks postoperatively, implant activation was undertaken by fitting the external audio processor. For the BB, a vibrogram was performed using the Connexx 6.4.3 and Symfit 6.0, which allowed direct testing of the hearing thresholds using the implant. Audiometric thresholds in free field and word recognition scores were determined and gain was adjusted at specific frequencies according to individual needs. Aided postoperative testing was done in soundfield, with the speaker at a 45-degree angle to the aided side, using a warble (frequency modulated) tonal stimulus, omnidirectional setting on microphone, with the good ear occluded and masked. In cases of bilateral implants, each ear is tested independently and in the bilateral condition. Patients were followed every 3 months whereby audiometric testing was again repeated and adjustments made to the device programming if needed. The average functional hearing gain (FHG) at 1 year was calculated comparing unaided free-field thresholds to thresholds with the device activated at 500, 1000, 2000, and 4000 Hz. At every audiological assessment, the status of the skin at the implant site was visually assessed. Any signs of erythema, edema, skin ulceration, or hair loss were documented.

Statistica (TIBCO Software, California, USA) was used for statistical analyses. For each study case in either group, paired sample t-tests were used to evaluate the difference between preoperative and the one-year postoperative mean audiometric threshold data (dB HL). Audiological preoperative and at the 1-year postoperative data were compared between the two groups using the Wilcoxon signed rank test for related samples and the Wilcoxon signed rank sum test for independent samples (with exact probability calculus), for p-values below 0.05. Effects of confounding variables (age, sex, type of hearing loss) were investigated by descriptive statistics.

## Results

### Clinical data

Demographic data is summarized in Table 1. A total of 18 BB and 8 AT patients met inclusion criteria and were included in this study. For the BB, the mean age was 10 years (range 5–16, SD 3.4 years), and 6 (33%) were females. A preoperative computed tomography (CT) scan was done in all patients, except for one where a magnetic resonance imaging (MRI) study was done as part of the hearing loss workup. The procedure was done on the right side in 13 cases (72%).

**Table 1:** Characteristics of patients treated with passive (*n* = 8) and active (*n* = 18) bone anchored hearing devices

Demographics	Transcutaneous bone anchored hearing device	
	Passive *n* (%)	Active *n* (%)
Age	8 years (range 6–13)	10 years (range 5–16)
Gender (M:F)	6 (75):2 (25)	12 (67):6 (33)
Implant ear (R:L)	3 (37.5):3 (37.5)	13 (72):5 (28)
Bilateral implants	2 (25)	0 (0)
Conductive hearing loss	8 (100)	17 (94)
Atresia	8 (100)	7 (39%)

In the BB cohort, the most common etiology was chronic otitis media (8 patients, 44%), followed by microtia/aural atresia (7 patients, 39%). Two had known ossicular fixation with associated CHL from prior middle ear exploration. One child had head trauma and subsequent CHL implicating ossicular involvement. All surgical placements were possible in the sinodural angle except for one whereby placement was supratemporal over the middle fossa dura. In this case, the CT scan demonstrated a severely sclerotic and contracted mastoid with anteromedial displacement of the sigmoid sinus.

The FMT was secured using 2-mm cortical bone fixation screws supplied by the manufacturer. In two cases, the supplied 2.4-mm emergency screws were used instead. None of the BB cases required placements of “lifts” for the FMT, as it was possible in all cases to achieve adequate depth for complete insertion. A degree of sigmoid sinus decompression was required in 9 cases (50%), and temporal dural decompression in 5 cases (28%). This sinus and dural decompression was minimal in all cases. No intraoperative complications were encountered. In two cases, both with microtia for which a staged microtia repair procedure was planned in the future, the incision was made further back just behind the hair line, instead of immediately behind the postauricular sulcus. In all cases, patients were sent home as same day surgery.

For the AT, the mean age was 8 years (range 6–13, SD 2.4 years) and two patients (25%) were females. Eight children comprised this cohort, two of whom had bilateral implants (bilateral conductive hearing loss) placed for a total of ten devices. All children had conductive hearing loss with congenital atresia being the most common etiology. The unilateral implants were split equally between the right and left ear in our patient sample. Aside from isolated ear canal atresia and microtia, there were two patients with unilateral hemifacial microsomia and one patient with chromosome mosaicism deletion syndrome (18q21 deletion).

For the AT group, the standard surgical approach recommended by the manufacturer was followed in all cases. No intraoperative complications were encountered. In all cases, patients were admitted for an overnight stay and then discharged home the following day. There was no statistically significant difference between the ages of the AT and BB groups. Throughout the study follow up period, all individuals in both cohorts remained compliant with daily use of their devices. Specific data on total hours of daily use was not gathered.

### Audiological outcomes

**Fig. 1:**
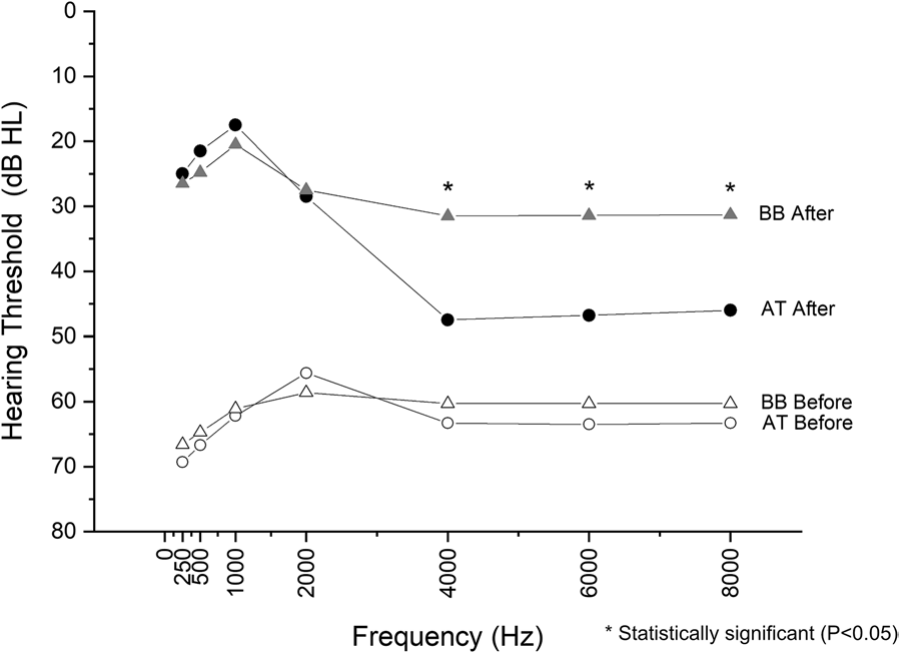
Pre- and post-operative hearing outcomes of air conduction thresholds between BB and AT

Pre and post implantation audiological data are summarized in Table 2 (AT) and Table 3 (BB). Both devices resulted in significant improvement in the hearing outcomes when comparing pre- and post-implantation thresholds (Fig. 1). There were no statistically significant differences in the functional gain between the two devices in the low and mid frequencies, with similar outcomes in free field assessment aided thresholds at 0.5, 1, and 2 kHz (BB 37.2 dB, AT 39.0 dB). However, the devices showed statistically significant differences in the improvements achieved at higher frequencies, with the BB resulting in significantly better hearing outcomes at 4, 6, and 8 kHz (28.9 dB vs. 16.7 dB). The increase in FG averages for the two groups between the low to mid (500–2000 Hz) and high (4000–8000 Hz) is summarized in Table 4.

**Table 2:** Audiological performance of the passive bone anchored hearing device (AT) before and after implantation

Frequency (Hz)	Before implant (*n* = 8)	After implant (*n* = 8)	Audiological gain
	dB HL (SD)	dB HL (SD)	dB
250	69.3 (16.6)	25.0 (8.2)	44.3
500	66.7 (15.2)	21.5 (8.5)	45.2
1000	62.2 (15.6)	17.5 (10.1)	44.7
2000	55.6 (13.8)	28.5 (19.1)	27.1
4000	63.3 (15.8)	47.5 (19.2)	15.8
6000	63.5 (16.1)	46.8 (19.7)	16.7
8000	63.6 (19.7)	46.0 (20.1)	17.6

**Table 3:** Audiological performance of the active bone anchored hearing device (BB) before and after implantation

Frequency (Hz)	Before implant (*n* = 18)	After implant (*n* = 18)	Functional gain
	dB HL (SD)	dB HL (SD)	dB
250	66.6 (17.7)	26.5 (4.8)	40.1
500	64.7 (20.5)	24.8 (8.3)	39.9
1000	61.1 (21.1)	20.5 (6.7)	40.6
2000	58.6 (22.6)	27.5 (6.0)	31.1
4000	60.3 (22.8)	31.5 (7.6)	28.8
6000	60.3 (22.5)	31.4 (7.7)	28.9
8000	60.3 (22.8)	31.3 (7.6)	29.0

**Table 4:** Average functional gain between the BB and AT groups for the low to mid (500–2000 Hz) and high (4000–8000 Hz) frequencies

	BB (dB)	AT (dB)	Difference (BB–AT, dB)	*P* value
Functional Gain (500–2000 Hz)	37.2	39.0	− 1.8	0.3461
Functional Gain (4000–8000 Hz)	28.9	16.7	12.2	< 0.0001

## Discussion

This is the first study looking at audiological outcomes between these two devices in the pediatric population. Our results demonstrated that indeed while both devices did well improving outcomes in the low and mid frequencies, the BB resulted in significantly better functional gain in the higher frequencies.

Many studies have noted high rates of soft tissue complications in children implanted with percutaneous BAHDs [7, 8]. This has translated to higher needs for revision surgeries and abutment changes often under repeat general anesthetic, as well as frequent and often unplanned physician visits [8–11]. Modifications to the surgical technique over the years has undoubtedly lowered these complication rates. However, aspects unique to the developing pediatric skull that can affect osseointegration [12], as well as confounding variables including higher rates of behavioural issues and the child’s reliance on a care provider, has meant that these challenges nevertheless remain a concern.

The main appeal of transcutaneous alternatives is the absence of a skin-penetrating abutment. Potentially, this could mean a lower risk of soft tissue complications and device loss from trauma, improved wear comfort, higher cosmetic acceptance by the patient, and elimination of the need for regular daily maintenance. Although there are more technical and surgical considerations for placement of transcutaneous BAHDs, studies over the past few years have shown these devices to be safe with favourable short term clinical outcomes [13–15]. In general, transcutaneous devices can be categorized as either passive or active. In the former, vibrations to the skull are driven by an external mechanical transducer, and the bone vibrations are sent via a magnetic connection to an internally implanted component, resulting in skull bone stimulation. With an active device on the other hand, the mechanical transducer is surgically implanted in the skull, and the externally worn component acts as a receiver transmitter. Given this design difference, there could be an audiological advantage to an active design in that it avoids the soft tissue attenuation of sound vibrations, with a 5–15 dB HL improvement in sensitivity at 1 kHz and above expected compared to a passive BAHD [16, 17]. It is also possible that the closer distance of the transducer to the cochlea could present an audiological advantage [18–20]. Furthermore, since the transducer is on the outside with a passive implant, a stronger magnetic connection may be required to improve audiological gain, and this may affect patient comfort.

The AT was introduced in 2013, and studies have since demonstrated this device’s safety and efficacy in the pediatric population, with significant audiological gain in the pure tone average at 0.5, 1, 2 and 4 kHz (PTA_4_) compared to the unaided condition. In a cohort of 10 children that received the AT system, Giannantonio et al. found a mean PTA_4_ functional gain of 23.70 dB [21]. Similarly, Powell et al. reviewed 12 patients (ten children and two adults) who received the AT, and noted a PTA_4_ functional gain of 30.2 dB. These studies however did not evaluate the FG in frequencies higher than 4 kHz. The current study likewise has demonstrated significant improvement in the low and mid frequencies, with a PTA_4_ functional gain of 33.2 dB.

From an audiological standpoint, the main potential advantage of the BB is avoiding soft tissue attenuation and hence a more favourable outcome in the higher frequencies. Studies thus have shown a mean functional gain ranging between 26.1 and 36.5 dB [22–26]. This functional gain is evident across all frequencies, although prior studies have suggested that it is more significant in the higher than in the lower frequencies [21]. This is in contradistinction to percutaneous BAHDs, where the functional gain appears to be more significant in the lower frequencies. Huber et al. [27] found that in human subjects, the thresholds improvement of transcranial attenuation (which is stronger at frequencies above 3 kHz [28]) was less in BB than in percutaneous BAHD recipients, suggesting better separation between ipsilateral and contralateral sides. In the current study, the BB demonstrated a PTA_4_ functional gain of 35.2 dB. When evaluating the gain at 6 and 8 kHz, the BB performed better than the AT (28.9 and 29.0 dB versus 16.7 and 17.3 dB respectively).

Zernotti et al. compared the BB to the Sophono (Medtronic; Boulder, Colo.), a passive transcutaneous BAHD, and found that both devices reduced air conduction thresholds at 1 and 2 kHz (*p* < 0.01), however only the BB device reduced the air conduction thresholds at 0.5 (*p* = 0.0140) and 4 kHz (*p* < 0.0001) post-implantation [29]. In this study, assessment of improvement at higher frequencies was unavailable. More recently, Han et al. evaluated audiological outcomes between three groups of adult patients who received either a percutaneous BAHD, the AT or the BB for MHL or SSD. In cases of MHL, there were no differences between the three groups in FG outcomes. However, the authors only measured hearing frequencies between 250 and 4000 Hz [30].

While the PTA_4_ has served as an important and standardized audiological outcome measure in data reporting, it disregards the higher frequencies from comparative analyses. In children, higher frequencies may play an important role in various aspects of speech and psychosocial development. The term minimal hearing loss has had various definitions in the pediatric population, and generally includes bilateral mild hearing loss, as well as high frequency hearing loss, in which the air-conduction thresholds are ≥ 25 dB HL at two or more frequencies above 2 kHz in both ears [31]. Historically, these children were not identified until they entered school, and even after that they often received little or no intervention. However, evidence gathered over time has demonstrated that children with minimal hearing loss face challenges in various domains including speech recognition, language development and competence, academic performance, psychosocial and emotional wellbeing, listening effort, and localization [32]. In the auditory rehabilitation of children with CHL and MHL, aiding the higher frequencies is an important component that is often under-evaluated in the assessment of the efficacy of implantable devices. The current study demonstrates the advantage of an active transcutaneous BAHD over a passive one in the higher frequencies in a consistent fashion in pediatric patients. Given what we know about the impact of high frequency hearing loss on child development, an active transcutaneous BAHD may provide long-term advantages in the pediatric population.

The BB does portray technical challenges given the relatively large dimensions of the implantable FMT. The general recommendation has been for surgical placement within the sinudural angle, although there are often limitations to complete fitting in this location within the pediatric population. While lifts are available ranging in size from one to four millimetres, a degree of dural decompression may be necessary. Alternatively, a retrosigmoid or supratemporal placement is an option [33, 34]. Rohani et al. have studied the potential impact of screw type, lift thickness, and FMT placement on sound transmission in BB surgery [35]. They found no significant differences in these factors to affect the vibartion of sound transmitted to the cochlea using Doppler vibrometry technique. You et al. have reviewed their experience with the supratemporal approach with BB surgery in forty patients and found the technique to be sae and effective [34]. Based on published data, we felt it was appropriate to include the patient who underwent supratemporal placement in our pooled data. The newly introduced BB 602 has addressed this technical limitation to some extent as its FMT is only 4.5 mm deep, which is approximately 50% that of the original BB 601 system used in this study. Early studies on the BB 602 have shown favourable clinical and audiological outcomes [36].

A limitation of our study is that primary outcomes of audiometric measures do not capture the broad utility of BAHDs. Quality of life (QOL) outcomes is not addressed and are a focus of future research.

## Conclusion

Both the AT and BB are transcutaneous BAHDs that are effective in the auditory rehabilitation of children with CHL and MHL. The devices are safe with a low complication profile and high patient and family acceptance. From an audiological standpoint, while both devices did well improving outcomes in the low and mid frequencies, the BB resulted in significantly better gain in the higher frequencies. Given the potential impact of high frequency hearing loss on child development, an active transcutaneous BAHD may provide long-term advantages in the pediatric population.

## Data Availability

All data for this study is securely stored on hard drives at the Principle Investigator’s clinical office. This data is available for review if requested.

## Abbreviations

AT: Cochlear attract
BAHD: Bone anchored hearing devices
BB: MedEl Bonebridge
CHL: Conductive hearing loss
CT: Computed tomography
FHG: Functional hearing gain
FMT: Floating mass transducer
MHL: Mixed hearing loss
MRI: Magnetic resonance imaging
PTA_4_: Four frequency pure tone average
